# Ageism Across Cultures and Interest in Geropsychology Among International Students

**DOI:** 10.1093/geroni/igaa057.1997

**Published:** 2020-12-16

**Authors:** Jessica Strong, Kirsten Graham

**Affiliations:** 1 University of Prince Edward Island, Charlottetown, Prince Edward Island, Canada; 2 Southern Utah University, Cedar City, Utah, United States

## Abstract

Ageist attitudes are concerning when considering who will enter the geriatric workforce. The impact of ageism on intent to work with older adults (OAs) between North American and non-North American individuals is unclear. We collected data from N=186 students (n=153 N. American, n=33 non-N. American), examining ageist attitudes and intent to work with OAs. We found significant differences between groups in ageist attitudes; North American students had more positive views of aging (M=88.64, SE=0.72) than non-North American students (M=85.33, SE = 1.42; t (167) = 2.04, p = 0.04, d=0.39), but there were no differences between groups for intent to work with OAs (t (174) = 0.09, p = 0.93). Ageist attitudes predicted intent to work with OAs for North American students only (F (2, 112) = 8.82, p < 0.001, R2 = 0.14). We discuss implications of ageism and intent to work with OAs from a cross-cultural lens.

